# Carl Woese: Still ahead of our time

**DOI:** 10.1002/mlf2.12049

**Published:** 2022-12-14

**Authors:** Patrick Forterre

**Affiliations:** ^1^ Institut Pasteur, Departement de Microbiologie Paris France; ^2^ Institute for Integrative Biology of the Cell, équipeBiologie Cellulaire des Archées, Département de Microbiologie Gif sur Yvette France

1

The work of Carl Woese and his colleagues of the Urbana University has been one of the most important breakthroughs in biology in the last century (for historical sketches, see Refs.^1^, ^2^). Carl Woese pioneered the use of macromolecule sequences to decipher the relationships between all organisms, fulfilling Darwin's dream to get a “*fairly true genealogical trees of each great kingdom of nature*”^3^. He went even beyond this objective since he was finally able to identify and connect each great kingdom of nature (which he called domains) into a single tree. The apex of his work was the discovery in 1977 of an entire new domain of life, hidden before our eyes, the Archaea^4^. The use of 16S ribosomal RNA (rRNA) as a molecular chronometer and later as a probe for PCR not only revealed a third domain of life but also paved the way for the detection and identification of microorganisms that were not yet amenable to cultivation^5^.

I was deeply affected when Carl Woese passed away 10 years ago because he was one of my three scientific heroes: together with James Watson, for the DNA double‐helix, and James Wang, for the discovery of DNA topoisomerases (the three Ws). I was not surprised but still shocked that despite his monumental achievements, his death was completely ignored in the French media, and I suspect that it was the same in most countries worldwide. Notably, Carl Woese died the same day as a famous horse, Ourasi, that had won several important competitions such as “le grand prix de l'arc de Triumphe.” The death of this horse made the headlines in most French media that day, including prime‐time TV news. I could not resist sending a short piece entitled “*the scientist and the horse*” to the French Journal Liberation in which I emphasize the difference in treatment between this horse (and I like horses) and one of the greatest scientists of the century. It was only published in the online version of the journal and the link has now been removed, but you can read it at the end of the fourth chapter of my book, *Microbes from Hell* ^1^.

Carl Woese was an ardent activist promoting the new vision of the living world revealed by his discoveries. He criticized to his death the misleading prokaryotic/eukaryotic paradigm, in which the classification of living organisms is based on phenotypic features, a sequel of the anthropic classification of living organisms between lower and higher ones, humans at the top of all classifications^6^. Notably, the prokaryotic/eukaryotic paradigm was establish only 15 years before the discovery of Archaea^7^. Therefore, when Carl Woese and George Fox published their seminar paper in *Proc Natl Acad Sci USA* in 1977, this paradigm was already firmly established. The prokaryote/eukaryote paradigm was easy to explain (with or without the nucleus) and quickly became the pillar of the classification of cellular organisms^8^. The prokaryote/eukaryote dichotomy was welcomed by cytologists and molecular biologists alike as the modern classification, to be opposed to the five‐kingdom classification favored by zoologists and botanists^9^. This probably explains why so many biologists, including molecular biologists, were strongly opposed at that time to Carl Woese's proposal even though it was based on molecular data^2^, ^10^, ^11^ (see Ref.^12^ for Carl Woese's answer to Ernst Mayer). Why should we change a winning team?

Here, I will briefly describe my various encounters with Carl Woese, focusing on our agreements and disagreements concerning the universal tree and the nature of the Last Universal Common Ancestor (LUCA), which, according to Carl Woese, was “*the most important and definitely less recognized major question in biology today*”^13^. I will also discuss how his legacy has been challenged on several occasions and his reactions. Unfortunately, Carl Woese is no longer among us to defend his legacy. All biologists aware of the existence of Archaea recognize the historical merit of Carl Woese in revealing to the world the existence of a deep divide in the “prokaryotic world,” but too many biologists (in my opinion) now believe that his view of the tree of life was fundamentally wrong and favor a pre‐Woesian evolutionary model. The prokaryote/eukaryote dichotomy is still the dominant one, paralleled by the virus/phage dichotomy in the virosphere, and the layman but also most politicians and journalists still ignore the existence of Archaea (still confused with Bacteria). More troublesome is the fact that most biologists (except those working on Archaea) are still unaware of their existence or vastly underestimate their importance. Therefore, it seems important to remind new generations of biologists of the importance of Carl Woese in the history of science and to fight for his ideas when we think that they are still at the forefront of life science.

## 2 THE EARLY FIGHT FOR THE MONOPHYLY OF ARCHAEA

**Figure 1:**
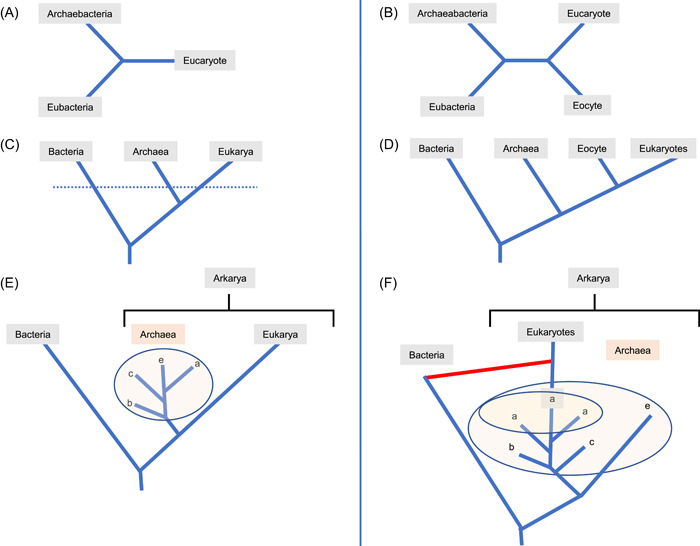
Parallel evolution of Woese's trees and Eocyte trees in the last four decades. (A) Schematic representation of the first unrooted tree based on rRNA. (B) The first unrooted Eocyte tree based on ribosome structure; in that tree, Eocytes (cells of dawn) included all thermophilic organisms, even those now included in Euryarchaeota, such as *Thermoplasma* and *Thermococcus*, in agreement with the hot origin of life hypothesis^18^. (C) The classical Woese's tree of life rooted in the “bacterial branch”^20^, with the dotted line corresponding to the Darwinian threshold^21^. (D) The eocyte tree rooted in the bacterial branch. *Thermoplasma* and *Thermococcus* were finally removed from Eocytes^33^. (E) The 3D tree based on RNA polymerase and a subset of large universal proteins^22^, ^23^. (a) Asgard archaea, (b) the BAT superphylum including *Bathyarchaeota*, *Aigiarchaeota*, and *Thaumarchaeota*, (c) *Crenarchaeota*, (e) *Euryarchaeota.* (F) The 2D tree based on various concatenations of universal proteins^24^, ^25^, ^26^. Eukaryotes branch within Asgard archaea (a). Archaea should include Eukaryotes to be monophyletic. Note that in both 2D and 3D trees, Arkarya^27^ are monophyletic. The red line indicates the association of a bacterium and an Asgard archaeon as the trigger of eukaryogenesis, producing for some a “ring of life” instead of a tree of life^28^. 2D, two‐domain; 3D, three‐domain; rRNA, ribosomal RNA.

I discovered Carl Woese's work on reading his 1981 review for *Scientific American* in which he described the discovery of “Archaebacteria” and the possibility of studying this new “*primary kingdom*” to determine the nature of the last common ancestor of the three domains, which he called the progenote^14^. This was the turning point in my career since I immediately stop working on DNA replication in Bacteria (*Escherichia coli*) and started working on DNA replication in Archaea, focusing on DNA topoisomerases and DNA polymerases. Thanks to the inspiration from Carl Woese and with the help of a few pioneers in the field, especially Wolfram Zillig^15^, I rapidly started to accumulate significant results from the study of *Halobacterium halobium* and *Sulfolobus acidocaldarius* ^16^, ^17^. Thanks to my first publications on the topic, I was invited by Wolfram Zillig to participate at the second meeting on “Archaebacteria” that he organized at the Martinsried Institute, near Munich in 1985. This gave me the opportunity to see Carl Woese for the first time. I vividly remember the memorable sparring between Carl Woese and James Lake that took place at this meeting. Based on electron microscopic pictures of ribosomes, James Lake had just published two papers in the *Proc Natl Acad Sci USA* and in *Science* ^18^, ^19^ in which he proposed replacing the three “urkingdoms” of life (Figure 1A) by four “urkingdoms” (Figure 1B). In his unrooted tree, based on ribosome structure, Archaebacteria were split between “Archaebacteria proper,” limited to halophiles and methanogens, and a new urkingdom, Eocytes, grouping *Sulfolobus* and other thermophilic archaea, including *Thermococcus* and *Thermoplasma* ^18^. James Lake, who spoke first, was later violently attacked by Carl Woese, who explained to him the different values of phenotypic versus genotypic data. Lake came back shivering without much argument in my opinion and most of the people present were in favor of Carl Woese explanation. The ribosomal structural signatures that Jim Lake used to define Eocyte were indeed rapidly also observed in the ribosome of a mesophilic methanogen, *Methanococcus vanielli* ^29^. Unfortunately, this paper, although published in *Science*, is no longer cited today, whereas Lake's paper published in 1984 is now cited by proponents of the two‐domains (2D) hypothesis (see below) as a historical argument supporting their favorite hypothesis^30^, ^31^.

James Lake was not discouraged by his ribosome failure and came back at the third meeting on Archaebacteria held in Victoria on the Canadian east coast with fresh arguments. In the meantime, he had learned phylogenetic techniques and designed a method of his own, evolutionary parsimony^32^, which still produced an unrooted four “urkingdoms” 16S rRNA tree, with *Thermoplasma* and *Thermococcus* removed this time from the Eocytes^33^. If the root of this tree is localized between bacteria and all other organisms, one indeed obtains 2D trees, and Lake's paper published in 1988 (Ref.^33^) should clearly be cited (instead of Lake's paper published in 1984) as the first description of a modern 2D tree (Figure 1D).

After each talk at the Victoria meeting, James Lake took the stage to conclude that the speaker has brought new arguments in favor of his theory. In striking contrast with the Munich meeting, Carl Woese was mute in Canada, leaving his coworker Gary Olsen to fight against James Lake^34^. Carl Woese seemed a bit discouraged at that time by the ongoing debate. How could people not realize that his scenario was the good one? I remember several colleagues wondering why a mathematician was not here to settle the dispute. I realized myself later that the problem was not primarily a mathematical one (today, people are speaking about how to choose the best model) but more a problem of data sets^22^. Carl Woese became fed up with meetings and was no longer eager to fight in person with his opponents. Besides the conflict with James Lake, I think that he was also deeply affected by the rejection of his theory by a few famous American evolutionists, such as Ernst Mayer and Lynn Margulis, who continue to support the prokaryote/eukaryote paradigm^2^.

## 3 THE FIRST STEP OF ARCHAEAL GENOMICS

My third encounter with Carl Woese was thus delayed until the third meeting organized by Wolfram Zillig in Munich in 1994, at the very beginning of the genomic era. Despite his reluctance to attend meetings, Carl Woese joined this one because of his friendship with Wolfram Zillig, who had been so influential in bringing the gospel of Archaea into Europe^15^. We were all waiting at that time for the first entire genome to be sequenced (most likely a bacterial one), and Roger Garret from Aarhus in Denmark^35^ organized a small roundtable with five other colleagues, including Carl Woese, to discuss the first (and probably the only one) archaeal genome that should be selected for this mammoth task. Each participant proposed the name of his favorite archaeon and when the floor went to Carl Woese, we all expected to get a good answer from him (hoping he would support our favorite one). “*We should sequence at least five,”* Carl Woese declared to our stupefaction and incredulity. In fact, Carl Woese was just right since five archaeal genomes were indeed available before the end of the century, and the first one by Craig Venter's team was done with his collaboration in 1996 (*Methanocaldococcus vannielii*)^36^. This was an example how it could be at the forefront of science in his lifetime.

## 4 THE EARLY CONTROVERSIES ABOUT THE ROOT AND LUCA

Despite Carl Woese being one of my heroes, I did not hesitate to disagree with him. Carl Woese was a strong proponent of rooting the universal tree of life between Bacteria and the two other domains, based on the phylogenetic work published by two groups at the end of the 1980s^37^, ^38^. This rooting was obtained by combining in a single phylogenetic analysis the universal trees of two paralogous proteins that originated by gene duplication before the LUCA. For me and my coworker at that time, Hervé Philippe, these trees were not reliable because of the attraction of the long bacterial branch in each of the two trees of paralogous proteins by the long branch that separated these two trees, corresponding to their respective outgroup^39^, ^40^. I suspected that Carl Woese preferred this rooting because it so clearly separated Archaea and Bacteria. Indeed, he used this rooted tree to justify changing the name Archaebacteria to Archaea^20^ and hoped that this topology would help other scientists to realize that Archaea were not simply new microorganisms living in extreme environments (Figure 1C).

For a while, I disagreed with Carl Woese because I preferred rooting the tree between Eukaryotes and the two other domains. This was because I liked the idea that some eukaryotic features, like the spliceosome, were possibly already present in LUCA. Of course, Carl Woese rejected this rooting since it could have been used to justify the old prokaryotic/eukaryotic division. When I published a paper with Hervé Philippe in which we defended this rooting by reporting the presence of more bacterial‐like genes than eukaryotic‐like genes in the first sequenced archaeal genomes^39^, Carl Woese ridiculed our argument as being quantitative but not qualitative, quality being the hallmark of the translation apparatus for evolutionary studies^41^. I soon realized that he was right, and I now support the bacterial rooting as allowing the most parsimonious scenario to explain the distribution of ribosomal proteins in the three domains of life^27^ (Figure 1E). However, I still think that the rooting problem cannot be solved by phylogenetic analyses but that a reasonable guess can be made from comparative molecular biology (Box 1).

Box 1. **Argument in favor of a simple LUCA**

LUCA had a smaller ribosome than modern organisms, with only the 33–34 universal proteins present in modern ribosomes that contain around 60–80 proteins^27^. The mechanism of ribosome biosynthesis in LUCA was much simpler, with a single universal protein being involved in this process^42^ **.** The initiation of transcription was less specific, with no transcription initiation factor being present in the universal protein set (the elongation first hypothesis of Finn Werner)^43^. The genome of LUCA was possibly still made of RNA, explaining the fact that the three major proteins involved in DNA replication, the replicase, the primase, and the helicase, are not homologous between Arkarya and Bacteria^44^ (Arkarya being the clade grouping Archaea and Eukarya)^27^. The few DNA replication proteins homologous between Bacteria and Arkarya (processivity factor) might have been introduced later independently in these two lineages by viruses. LUCA probably had no ATPase synthase and produced ATP by fermentation since the ATP synthase subunit essential for ATP synthesis is not homologous between Bacteria and Arkarya^45^.

These arguments are based on parsimonious reasoning and Carl Woese's idea that sophistication of fundamental molecular mechanisms takes place independently in the three major lineages. Alternative hypotheses (complex LUCA) involve the replacement of the Arkaryal features present in LUCA by bacterial ones or vice versa the replacement of bacterial features present in LUCA by Arkaryal ones. There is no obvious selection pressure suggested for this replacement, except possibly simplification in the bacterial branch^40^. Although LUCA was probably simpler than modern organisms, it was probably not a progenote, *sensu* Woese and Fox^21^, ^46^, since it already has a sophisticated membrane with protein pumps and is capable of faithful protein synthesis^27^.

In their other seminal 1977 paper “*The concept of cellular evolution*”^46^, Carl Woese and George Fox suggested that LUCA still had an RNA genome and an unfaithful translation apparatus. Again, today, an RNA LUCA is the most parsimonious hypothesis to explain the major gap that exists between the molecular biology of Bacteria on the one hand and the molecular biology of Archaea and Eukarya on the other (Box 1). Of course, evolution is not always parsimonious, so we should remain open to possible alternatives^47^. If I partly agree with Carl Woese's view about LUCA, I do not think that LUCA was a progenote, *stricto sensu*, because it was probably already able to translate the mRNA message rather faithfully. This conclusion came from the fact that LUCA probably already contained several proteins involved in transfer RNA (tRNA) modifications that are crucial for faithful translation. A good example is provided by the two universal proteins Kae1 and Sua5 that are essential for t6A biosynthesis, a universal tRNA modification essential for the reading of ANN codons. Although these two proteins need additional factors to perform their task in the three domains, they are sufficient to synthesize t6A in mitochondria, suggesting that t6A biosynthesis in mitochondria recapitulates t6A biosynthesis in LUCA^27^.

## 5 THE DARWINIAN THRESHOLD AND THE EVOLUTIONARY TEMPO

Although Carl Woese adopted and promoted the universal tree rooted in the so‐called “bacterial branch,” he never considered finding a name for the clade grouping Archaea and Eukarya. I suspect that this departure from cladistic rules was due to his fear of weakening the three‐domain concept that he first promoted. Indeed, if one follows cladistic rules, Woese's tree is not a three‐domain tree, as is usually assumed, but a two‐domain tree, one being Bacteria, the other being the clade grouping Archaea and Eukarya that I suggested naming Arkarya^27^. Probably to justify bypassing cladistic rules in the case of the universal tree, in 2002, Carl Woese introduced the concept of the Darwinian threshold to describe the transition period between LUCA and the ancestors of the three domains (the Last Archaeal Common Ancestor, LACA; the Last Bacterial Common Ancestor, LBCA; and the Last Eukaryotic Common Ancestor, LECA), hereafter referred to as the three ancestors^21^. For Carl Woese, the Darwinian threshold is the moment when the transmission of genetic information moves from a predominantly horizontal mode based on lateral gene transfer (LGT) to a predominantly vertical mode. He assumed that evolution was not Darwinian before the threshold because LGTs were so dominant that they do not allow classification of organisms. Accordingly, one can argue that the cladistic rules were not applicable before the threshold, including at the time of the separation between the lineages leading to Archaea and Eukarya (Figure 1C).

Killing two birds at once, Carl Woese also introduced the Darwinian threshold to explain why the tempo of evolution dramatically decreased between the time of LUCA and the emergence of the three ancestors. This dramatic reduction in the evolutionary tempo was one of the major observations made by Carl Woese very early on by looking at the shape of the rRNA tree^13^. He realized that if the rate of rRNA sequence evolution within the domain was extrapolated to the time between LUCA and the tree ancestors, LUCA was born before the formation of the Earth! However, explaining the reduction of the evolutionary tempo simply by a dramatic reduction in LGT prevalence was probably not correct since this reduction of the evolutionary tempo between LUCA and the three ancestors also takes place in the evolution of universal proteins that are very rarely affected by LGT^48^, ^49^. In 2006, I suggested that this reduction in the evolutionary tempo might have been due to the transition from RNA to DNA genomes since DNA can be replicated and repaired more faithfully than RNA^50^. I introduced viruses in the scenario as triggers of three independent RNA‐to‐DNA transitions, one for each domain. To my delight, Carl Woese did not reject this hypothesis and agreed to refer my paper to the *Proc Natl Acad Sci USA*.

I think that Carl Woese also proposed the Darwinian threshold to respond to those critics who used LGT at the end of the last century to challenge the tree of life. The discovery that LGT between organisms has been more prevalent than once thought, sometimes even between organisms far apart in evolutionary trees, led to a buzz suggesting forgetting about the tree all at once and trumpeting that “*Darwin was wrong*” at least for microorganisms (for a review and discussion of this topic, see Ref.^51^). Carl Woese was profoundly affected by these views, which led some authors to jokingly refer to his tree as “*the tree of one percent*”^52^ or suggested replacing trees by networks^53^. The Darwinian threshold was his way to save the upper part of the universal tree (those with three domains) in this context.

## 6 MEETING CARL IN PERSON

In the meantime, I not only had the chance to meet Carl Woese at another meeting in 2003 but also have personal discussions with him about evolution. I was a little bit afraid that his knowledge of my previous disagreement with his favorite rooting could have a negative impact on our encounter. This was not the case; Carl Woese was immediately friendly, possibly because he realized that I was a real lover of archaea, something more important to him than disagreement about the topology of the universal tree. I was invited to give a talk on the molecular biology of Archaea, and Carl Woese was happy that I introduced my talk with a figure from his *Scientific American* paper in 1982, surprised that someone remembered this review^14^. The 2003 meeting was organized at Lund in Sweden to honor Carl Woese when he was awarded the Crafoord prize by the Royal Swedish Academy of Sciences. Carl Woese received this prize from the King of Sweden, Gustav XVI, a descendant of the French Marshal of Napoleon Bernadotte, an unexpected turn in the world history. I discovered on this occasion the humor of Carl Woese when he mimicked at the official dinner the ritual of the master of ceremony (see Chap. 4 in Ref.^1^).

The Crafoord prize, awarded only once in 3 years to biologists, is as important as the Nobel prize (both in terms of money and ceremony!), the only exception being the complete absence of journalists and press coverage. It is a pity that Carl Woese never received the Nobel Prize. This would have been an opportunity for the press to speak about archaea. Carl Woese clearly deserved the Nobel prize in medicine, considering the impact of the use of molecular markers for microorganism identification in medicine. The recent Nobel prize awarded to Svante Pääbo for the sequencing of Neanderthal genomes confirms that great advances in the fields of evolution indeed merit the Nobel prize. I wonder if the opposition of his adversaries among American evolutionists played a role in the fact that Carl Woese never received this final distinction.

The last time I met Carl Woese in person was in 2007 on the occasion of the meeting held at the University of Illinois in Urbana‐Champaign to celebrate the 30th anniversary of his discovery of Archaea. The meeting was entitled “*hidden before your eyes*” since Archaea, resembling Bacteria, were indeed visible, but confused for decades with their cousins under the microscope. This was the opportunity for me to visit his laboratory and I had the chance to see Carl Woese opening the box containing the film of the two‐dimensional gel chromatography corresponding to the collection of 16S rRNA oligonucleotides that he obtained after digestion of the 16S rRNA of *Methanobacterium thermoautotrophicum* by the RNase T1 (Figure 1 in Ref.^15^). Carl Woese was happy to be honored at his own university and to be surrounded by so many scientific friends who were among the first to introduce Archaea in various countries, such as Karl Stetter in Germany, Yoshizumi Ishino in Japan, and David Prangishvili in the former USSR.

## 7 CHALLENGES TO CARL WOESE'S LEGACY

Carl Woese passed away in 2012, before the reawakening of the James Lake Eocyte tree with the discovery of Asgard archaea^24^, ^25^. I am really wondering how he would have reacted to this new twist in the history of biology. When I remember his fight with James Lake, I imagine that he would have been deeply disturbed, especially by the fact that very few scientists still support his view. In the 2D trees published in the last decade, Archaea became paraphyletic, since Eukarya branch within Archaea, except if one considers that Eukarya belong to the Archaea (Figure 1F). To make things worse, the modern Eocyte trees are often depicted as fusions between one or two bacteria (one being the ancestor of mitochondria) and one archaeon, much like in the scenario promoted by Lynn Margulis and highly despised by Carl Woese (figure 1 in Ref^14^). In his review published in 1981 about Archaebacteria, Carl Woese hence illustrated the impact of his discovery by contrasting his new tree with an old one in which eukaryotes emerged from the fusion of several prokaryotes (only Bacteria at that time)^14^. This was the figure that I used in my talk at Lund and that Carl Woese liked so much. It seems now that we are going back to the past with the old tree becoming the new one.

In fact, the classical Woese's tree is most likely still the correct one (Figure 1E). The 2D trees obtained during the last decade can be explained by the presence of fast‐evolving species and small proteins in data sets, combined with the difficulty in recovering the short branch testifying to the monophyly of Archaea (for reviews and recent results, see Ref.^54^). This does not mean that the discovery of Asgard archaea was not a seminal breakthrough since these Archaea indeed provide exciting new data to understand the emergence of Eukarya. Phylogenetic analyses revealed that the odd distribution of some Eukaryotic Signature Proteins (ESPs) was most likely due to extensive LGT between Asgard and proto‐eukaryotes^54^. Studying these proteins will thus help us to reconstruct their history in the proto‐eukaryotic lineage. The extensive LGT that we observed between Asgard archaea and proto‐eukaryotes also suggests that these organisms were thriving together for a long time before LECA in the same environments, indicating that study of the Asgard archaea should shed light on the type of environment inhabited by our proto‐eukaryotic ancestors. The first cultivated Asgard archaeon, Candidatus Prometheoarchaem synthrophicum, turned out to be dependent for growth on its physical association with a methanogenic archaeon^55^. Notably, the authors of this tour de force concluded in their publication from comparative genomics of various Asgard lineages that “*most of them, if not all, should be dependent of symbiotic interactions.*” It is thus tempting to suggest that LGT between proto‐eukaryotes and Asgard was facilitated by symbiotic interactions between proto‐eukaryotes and Asgard^54^. One can even imagine that some Asgard archaea still live as ectosymbionts of Eukarya today. The search for such associations in modern biotopes could be an exciting adventure for young (or less young) microbial ecologists^54^.

In their assault against the three‐domains concept, some authors went as far as to deny the prokaryotic split between Archaea and Bacteria. At the beginning of 2020, a publication was heralded by some as putting the final nail in Carl Woese's coffin. From the analysis of 10,575 genomes, 381 protein markers, and “*1.16 trillions non gap amino‐acids*,” the authors concluded that the branch that separates Archaea and Bacteria was much shorter than previously thought^56^. In their published tree, this branch was indeed shorter than the branches separating some bacterial groups altogether. If this was correct, there was no longer a reason to separate Archaea from Bacteria, prokaryotes being reunified in one domain, as in the pre‐Woese area. Looking even cursorily at the data supporting this claim, it was obvious that there were many flaws in this conclusion. The authors only included 6 of the 34 ribosomal proteins shared between the two domains in their data set of 381 proteins. They also included in their data set proteins that have been affected by LGT between Archaea and Bacteria. This is, for instance, the case of DNA gyrase, which is a bacterial protein recruited by some Archaea (Ref. ^50^ and references therein). This paper was indeed subsequently refuted by Moody and colleagues, who identified both LGT and hidden paralogy in the data set of Zhu and colleagues^49^. They noticed that 14 trees include no archaea, something *a priori* unbelievable when the aim was to measure the distance between the two domains, and that 68 others contain less than 25% of the sampled archaea. Strikingly, the monophyly of Archaea and Bacteria (a prerequisite for this kind of analysis) was only recovered in 22 of the 381 published trees^49^. This indicated that, as in the case of DNA gyrase, most of the 381 proteins were not present in LUCA but were transferred by LGT from one domain to the other.

The long branch between Archaea and Bacteria first observed by Carl Woese in the rRNA tree was in fact also recovered long ago in universal protein trees (for a review, see Refs.^23^, ^57^) and confirmed recently by several authors using extensive genomic data, both for ribosomal and for non‐ribosomal proteins^22^, ^48^, ^49^. Long branch lengths between these two domains should be a major criterion in determining which universal protein was indeed present in LUCA, besides widespread distribution within each of the two domains^48^, ^58^. Failure to consider this argument can led to wrong identification of LUCA proteins as observed in the work of Weiss and colleagues^59^. The authors reported the identification of 355 proteins that were supposed to be present in LUCA, starting from “6*.1 million protein‐coding genes*” and “*286,514 protein clusters.*” They concluded that LUCA showed a metabolism very similar to those of some Clostridia or methanogens, and still had a DNA genome, resembling more a modern “prokaryote” than a progenote. They also concluded that LUCA thrived at high temperatures because reverse gyrase, an enzymes that is not found in mesophiles but is essential for hyperthermophiles, was present in LUCA. Archaeal and bacterial reverse gyrases indeed formed two monophyletic groups in their analysis, but the branch separating them was very short, and later work based on more reverse gyrase sequences recovered a tree in which archaeal and bacterial reverse gyrases were intermixed, suggesting LGT of this protein between these two domains^58^. A complete reanalysis of Weiss and colleagues' data by Berkemer and McGlynn revealed that about 82% of the 355 proteins used in their analysis produced trees with very short branches between the two domains (or no branch at all), suggesting that they were not present in LUCA^48^. Archaea were again underrepresented in many trees with very low diversity. I have also looked at the data and arrived at a similar conclusion. Using as criteria the fact that Archaea and Bacteria should be separated by long branches and that both domains should be represented by a reasonable collection of distantly related phylums, I found that only 15–20 of the 355 “LUCA” proteins in the data set of Weiss and colleagues were probably present in LUCA (unpublished observations). One can conclude that the proteome of LUCA is still on the table.

One can wonder why the papers supporting fusion scenarios between Archaea and Bacteria for the origin of eukaryotes or claiming that the branch between Archaea and Bacteria is vanishing were published in first‐rate journals and received substantial press coverage. This is probably because they perpetuate the prokaryote/eukaryote paradigm, which remains so precious to many biologists. The term prokaryote is still widely used and some authors even continue to use the term archaebacteria. Unfortunately, the confusion will remain with the new nomenclature proposed in the Genome Taxonomy DataBase (GTDB) since they include “bacteria” in the name of two major new archaeal phyla “Halobacteriota” and “Methanobacteriota”^60^. Notably, this is based on the strict rules of the International Code of Nomenclature of “Prokaryotes” that still does not discriminate between Archaea and Bacteria.

In my opinion, the renewal of the prokaryote/eukaryote paradigm is an unfortunate byproduct of the genomic and metagenomic era. Archaeal and bacterial genomes are indeed very similar (small, compact, organized in operon, etc.) and strikingly different from the eukaryotic genomes. Large proportions of the archaeal mobilome (for instance, conjugative plasmids or head‐and‐tailed “phages”) are also very similar in the two “prokaryotic” domains. The previous focus on the translation apparatus and other major mechanisms involved in the expression and transmission of the genetic information thus faded away. A corollary of the genomic and metagenomic expansion has been the invasion of biology by big data. Big data can be extremely useful of course but they are also fashionable, as indicated by expressions such as “*1.16 trillions non gap amino‐acids*” or “*286,514 protein clusters.”* This focus on big data has favored a holistic view of the biosphere, which is only positive if it does not replace quality by quantity in data analyses.

Another unfortunate consequence is that many biologists now again underestimate the importance of the reductionist methodology in science. The work of Carl Woese has been one of the many triumphs of the reductionist approach in biology since he was able to unravel the major division of the living world (apart from viruses) using a single gene, the 16S rRNA^61^. This division in three domains has been validated later by comparative genomics using dozens of universally conserved genes present in LUCA^23^. The use of universal proteins obtained from genome sequencing increased our knowledge of the topology of the tree of life, revealing the long branch of bacteria and the division into two primary domains: Bacteria and Arkarya^27^. This is still a triumph of reductionism since recovery of the correct tree can be done with a single protein, the RNA polymerase, and/or by a bunch of correctly selected universal proteins^22^ (Figure 1E). This was also a triumph of molecular biology since discovery of the major division of the living world and the topology of the tree was based on the dramatic advances made in the last century in this discipline.

Paradoxically, Carl Woese fiercely attacked molecular biology and “reductionism” in several of his later publications. He criticized molecular biology because “*molecular biology, the dominant biological, discipline of the time, did not even recognize the evolutionary process as a scientific problem*”^6^ which was indeed true for many biochemists and geneticists who were happy with the simple concept that “pro”karyotes preceded eukaryotes and shared a mechanistic view of the biological world. Notably, I obtained a degree in molecular biology at the University of Paris in 1971 with a lot of lectures on biochemistry and genetics but not a single lecture on evolution. However, molecular biology offered Carl Woese both the 16S rRNA tool and the translation apparatus as fundamental evolutionary markers. As famously claimed by Theodosius Dobzhanski “*Nothing in Biology Makes Sense Except in light of Evolution*”^62^, and the translation apparatus is no exception. To put molecular biology back on its feet, it is sufficient to say that the aim of this discipline should be to retrace the history of the emergence and evolution of molecular machines and their connections. In that sense, one can claim that Carl Woese indeed put molecular biology back on its feet.

Molecular biology without evolution was criticized by Carl Woese as reductionism in action. This was a right criticism of reductionist metaphysics that translates a methodological approach into a broad view of the world, with some microbiologists “*treating microorganisms effectively as bags of interesting biochemistry*”^6^. However, one should not “throw the baby out with the bathwater” in bypassing the reductionist step in the process of scientific discovery. The discovery of Archaea based on single‐gene analyses has been a triumph of methodological reductionism^61^. The scientific process should be a combination of reductionist and holistic approaches.

The present underestimation of the reductionist methodology is well illustrated by the difficulty in recovering raw data from recent papers. This is especially the case for published trees based on protein concatenation and analyses based on a combination of multiple trees, since the individual trees are often no longer published in supplementary materials. This makes examination of a single tree, a critically important reductionist task, a difficult and time‐consuming task for people who wish to reproduce and check the conclusion of comparative genomics or phylogenetic analysis papers. The marker selection process is a critical step in phylogenetic analysis since a single misleading protein can completely change the result obtained by the concatenation of dozens of proteins. This has been shown, for instance, in the case of elongation factor EF2 of two lineages (out of 18) of Asgard archaea that did not branch with other Asgard EF2 but as a sister group of eukaryotes, probably because of ancient LGT between these Asgard and proto‐eukaryotes^22^, ^54^. Removal of this protein from the original data set of 36 markers dramatically changed the topology of the final tree, producing a 3D tree in the absence of fast‐evolving species^22^. Conversely, addition of a single eukaryotic protein of bacterial origin in a data set of 29 protein markers can transform a 2D tree into a 3D tree, even in the presence of fast‐evolving species^26^. Notably, examination of single trees was essential to identify the factors that favor the 2D topology in the final concatenation: small proteins, fast‐evolving species, and hidden LGT^22^, ^54^ or else in the refutation of the papers that underestimated the importance of the branch length between Archaea and Bacteria^48^, ^49^.

## 8 CONCLUSION

Three years before his death, Carl Woese wrote: “*the ‘prokaryote era’ is fast drawing to a close, and many microbiologists (especially the younger ones) are no longer structured in their thinking by the prokaryote notion*”^6^. Unfortunately, this was too optimistic since the prokaryotic notion is again on the rise and the Eocyte (Asgard) scenario is now the new paradigm. Most scientists working on Archaea work within the framework of this paradigm, even when the data move in the other direction. For instance, whereas Asgard viruses detected are typical for archaeal viruses, some authors emphasize the eukaryotic features of Asgard *Caudoviricetes* (head and tailed viruses), although these features are also present in some bacterial *Caudovircetes* ^63^, ^64^. Interestingly, Jüttner and Ferreira‐Cerca recently reported that all Asgard archaea, except Odinarchaea, have unlinked tRNA genes^65^. This strongly suggests that Asgard probably cannot be the ancestor of eukaryotes since, according to these authors, “*there is no known natural example of organisms with linked rRNA genes deriving from an ancestor with unlinked rRNA genes*,” and Eukarya were never grouped with Odinarchaea in 2D phylogenies. However, the authors only proposed evolutionary scenarios in which Eukarya originated from a hypothetical Asgard with linked rRNA genes, based on the quantitative argument that “*most existing phylogeny analyses placing the eukaryotic lineage within the Asgard archaea*.” Notably, Jüttner and Ferreira‐Cerca also reported that unlinked rRNA genes are also frequent in DPANN and that “*unlinked rRNA genes are more often found in symbiotic organisms*”^65^. In my opinion, the existence of unlinked rRNA genes in practically all Asgard archaea is thus another argument supporting the idea that most of them are symbiotic organisms.

In conclusion, I think that most of Carl Woese proposals turned out to be right; LUCA was most likely a much simpler organism than modern organisms (although not as simple as he suggested) and the living world, except viruses, can be adequately represented by a trinity: Archaea, Bacteria, and Eukarya. The tree of life topology preferred by Carl Woese, with Archaea and Eukarya being sister groups, is most likely the right one, even if this is not yet recognized today. In the summary of this essay, I wrote that most of Carl Woese's ideas will probably be recognized in the future. This means that Carl Woese is still ahead of our time. This is possibly an optimistic view, but I really think that our present knowledge in molecular biology and critical analysis of already published phylogenetic data are sufficient to bet in favor of Woese's tree. In a recent perspective, we provide advice that could be useful for future phylogenetic work designed to build a universal tree (Box 1 in Refs.^22^, ^47^). I really hope that more evolutionists will take the challenge with open eyes, as Carl Woese himself did in his youth.
