# Correction to “Carl Woese: Still ahead of our time”

**DOI:** 10.1002/mlf2.12166

**Published:** 2025-03-27

Forterre, P. Carl Woese: still ahead of our time. *mlife*. 2022;1:359–367. https://doi.org/10.1002/mlf2.12049

In the Conclusion section, the text “all Asgard archaea, except Odinarchaea, have unlinked tRNA genes” was incorrect. This should be replaced by “all Asgard archaea, except Odinarchaea, have unlinked rRNA genes.” The text “(Box 1 in Refs^22,47^)” was incorrect. This should be replaced by “(Box 1 in Refs^54^)”.

I apologize for these errors.

